# Macrophage-mediated anti-tumor immunity against high-risk neuroblastoma

**DOI:** 10.1038/s41435-022-00172-w

**Published:** 2022-05-07

**Authors:** Xao X. Tang, Hiroyuki Shimada, Naohiko Ikegaki

**Affiliations:** 1 Department of Anatomy and Cell Biology, College of Medicine, University of Illinois at Chicago, Chicago, IL 60612 USA; 2 Departments of Pathology and Pediatrics, School of Medicine, Stanford University, Stanford, CA 94305 USA

**Keywords:** Tumour immunology, Immunotherapy

## Abstract

Neuroblastoma is the most common extracranial childhood solid tumor. The majority of high-risk neuroblastoma is resistant/refractory to the current high intensity therapy. Neuroblastoma lacks classical HLA Class I expression and exhibits low mutation burden, allowing neuroblastoma cells to evade CD8+ T cell-mediated immunity. Neuroblastoma cells do not express PD-L1, and tumor-associated macrophages are the predominant PD-L1+ cells in the tumor. In this study, we performed gene expression profiling and survival analyses on large neuroblastoma datasets to address the prognostic effect of *PD-L1* gene expression and the possible involvement of the SLAMF7 pathway in the anti-neuroblastoma immunity. High-level expression of *PD-L1* was found significantly associated with better outcome of high-risk neuroblastoma patients; two populations of *PD-1*+ *PD-L1*+ macrophages could be present in high-risk tumors with *PD-1/PD-L1* ratios, ≈1 and >1. Patients with the *PD-1*/*PD-L1* ratio >1 tumor showed inferior survival. High-level co-expression of *SLAMF7* and *SH2D1B* was significantly associated with better survival of the high-risk neuroblastoma patients. Together, this study supports the hypothesis that macrophages are important effector cells in the anti-high-risk neuroblastoma immunity, that PD-1 blockade therapy can be beneficial to the high-risk neuroblastoma subset with the *PD-1/PD-L1* expression ratio >1, and that SLAMF7 is a new therapeutic target of high-risk neuroblastoma.

## Introduction

Neuroblastoma is the most common extracranial childhood solid tumor with two distinct types: favorable and unfavorable. The majority of high-risk neuroblastomas are unfavorable tumors that are resistant/refractory to the current high-intensity therapy, and the survival of patients with the high-risk tumors remains poor [1]. Immunotherapy would be a promising approach against high-risk neuroblastoma, but it has its own challenge because of the limited immune cell types to deliver their cytotoxicity against the malignant tumor cells in an antigen-specific manner. Neuroblastoma lacks expression of classical HLA Class I [2, 3], allowing the tumor cells to evade CD8+ T cell-mediated immunity. In addition, neuroblastoma exhibits low mutational burden [4], further restricting the availability of tumor-specific T cell clones. CD8 CTLs are thus expected to play little role in the anti-neuroblastoma immune response. Our previous study suggests that CD4 CTLs are important effector cells against high-risk neuroblastoma, but the CD4 CTL mediated-“protective effect” declines over time, in part due to the progressive formation of an immunosuppressive tumor microenvironment (TME) [5] and/or chemotherapy-induced suppression of hematopoiesis [6].

In this study, we performed gene expression profiling and Kaplan-Meier survival analyses on two independent neuroblastoma datasets to address clinical relevance of tumor-infiltrating macrophages for the disease. The gene expression analysis was chosen for this study, because the limited availability of human neuroblastoma specimens has made virtually impossible to perform a live cell-based analysis (e.g., CyTOF or single-cell RNA-seq) on a large cohort of human neuroblastomas to evaluate an anti-tumor immune response.

We first focused on the prognostic significance of *PD-L1* expression in neuroblastoma, for which the results have been inconsistent among different research groups [7–12]. PD-L1 is an important immune checkpoint, and it is constitutively expressed on macrophages [13, 14]. PD-L1 expression is also found on other immune cells (dendritic cells, B cells, T cells) [14] and some tumor cells [15]. However, based on our multiplex immunohistochemistry (IHC) analysis, neuroblastoma cells do not express PD-L1, and PD-L1+ cells detected in neuroblastoma tissues are predominantly macrophages [5]. In fact, this study revealed that high-level expression of macrophage-derived *PD-L1* was significantly associated with better patient outcome, including those with high-risk neuroblastoma.

PD-L1 is a ligand of the inhibitory receptor PD-1, and the engagement of PD-L1 on PD-1 negatively modulates the function of PD-1+ cells [16]. It has been shown that tumor-associated macrophages (TAMs) co-express PD-1 and PD-L1, and the PD-1/PD-L1 signaling in macrophages impairs their phagocytic capacity [17–19]. Therefore, it was of interest to address clinical significance of the *PD-1* expression in relation to *PD-L1* expression in high-risk neuroblastomas. We have found that two populations of *PD-1*+*PD-L1*+ macrophages could be present in high-risk neuroblastoma tissues with *PD-1/PD-L1* ratios, ≈1 and >1 phenotypes. Patients bearing tumors with the *PD-1*/*PD-L1* ratio >1 showed inferior survival.

We have also explored clinical relevance of the SLAMF7 pathway in the anti-high-risk neuroblastoma immune response. SLAMF7 is a transmembrane receptor present on macrophages and other immune cell types [20, 21]. SLAMF7 functions as homotypic receptor, and its transduction-signaling pathway is triggered by the interaction between neighboring cells (trans-interaction) or on the same cell (cis-interaction). Upon homotypic interaction, SLAMF7 recruits the SAP cytoplasmic adaptor EAT-2, encoded by *SH2D1B* [22], to its tyrosine-phosphorylated ITSM [23]. This results in the activation of macrophage phagocytosis [24]. In contrast, SLAMF7 activation in the absence of EAT-2 results in cellular inhibition via recruitment of inhibitory phosphatases (SHP1, SHP2, SHIP1, and csk) [23]. Our results show that high-level co-expression of *SLAMF7* and *SH2D1B* is significantly associated with better outcome of high-risk neuroblastoma patients. This observation suggests that activation of the SLAMF7 pathway is an important biological process in the macrophage-mediated anti-tumor immunity against high-risk neuroblastoma.

Collectively, our data support the hypothesis that macrophages are important effector cells in the anti-high-risk neuroblastoma immune response, that PD-1 blockade therapy can be beneficial to the high-risk neuroblastoma subset with the *PD-1/PD-L1* ratio >1, and that SLAMF7 is a promising new therapeutic target of high-risk neuroblastoma. We further discuss therapeutic interventions to maximize tumor phagocytosis function of macrophages against high-risk neuroblastoma.

## Results

### TAMs as the predominant source of classical HLA Class II expression in high-risk neuroblastoma tissues

**Fig. 1:**
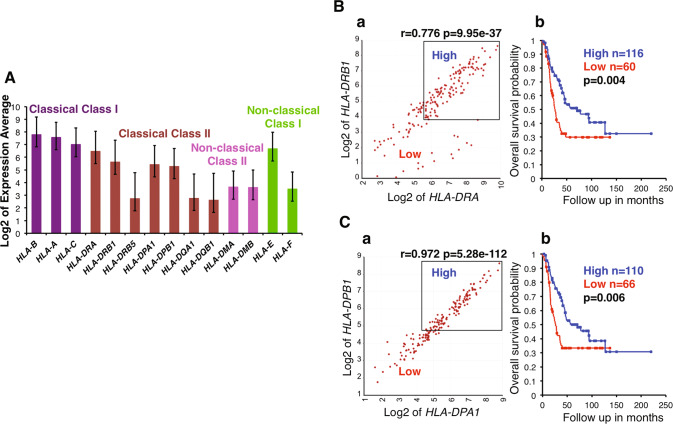
High-level expression of classical HLA Class II genes in high-risk neuroblastoma and its clinical implications. **A** HLA-related gene expression was examined for the high-risk subset of Cohort 1 by the R2. High-level expression of classical HLA-Class II genes (*HLA-DRA, HLA-DRB1, HLA-DPA1, HLA-DPB1*) was detected in high-risk neuroblastoma tissues. A similar trend was observed in Cohort 2 (see Fig. S1). **B** (**a**) *HLA-DRA* expression was significantly correlated with *HLA-DRB1* expression in high-risk neuroblastomas of Cohort 1. (**b**) Combination of high *HLA-DRA* and *HLA-DRB1* expressions was significantly associated with prolonged survival of the high-risk patients of Cohort 1. **C** (**a**) *HLA-DPA1* expression was significantly correlated with *HLA-DPB1* expression in high-risk neuroblastomas of Cohort 1. (**b**) Combination of high *HLA-DPA1* and *HLA-DPB1* expressions was significantly associated with prolonged survival of the high-risk patients of Cohort 1. The expression unit of genes in Cohort 1 is Reads Per Million (RPM). Average expression of each gene was shown as Log2 of RPM. Error bars indicate standard deviations.

To gain a better insight into the overall immune cell landscape of high-risk neuroblastoma, we examined the pattern of HLA-related gene expression. As shown in Fig. 1, high levels of classical HLA Class I genes and Class II genes were detected in high-risk neuroblastoma tissues. It is well established that neuroblastoma cells do not express classical HLA Class I and II [2, 3], and that stromal cells and tumor-infiltrating immune cells expressed classical HLA Class I, and tumor-associated macrophages, dendritic cells and B cells express classical HLA Class II molecules in neuroblastoma tissues [3, 25–27]. Therefore, Fig. 1 suggested that the expression of classical HLA Class I genes originated from the stroma and tumor infiltrating immune cells, and that the high expression of the classical HLA Class II genes must be derived from the immune cells, including tumor-associated macrophages (TAMs), dendritic cells and B cells [3, 26, 28, 29].

To address the clinical effect of the HLA Class II positive cells on neuroblastoma cells, we examined the effect of *HLA*-*DRA*, -*DRB1*, -*DPA1* and -*DPB1* expressions on survival of high-risk neuroblastoma patients. As shown in Fig. 1B, C, high expressions of the HLA class II genes in their appropriate combinations were significantly associated with prolonged survival of the patients. These observations support the hypothesis that HLA Class II positive cells are involved in the anti-high-risk neuroblastoma immune response at diagnosis.

**Fig. 2:**
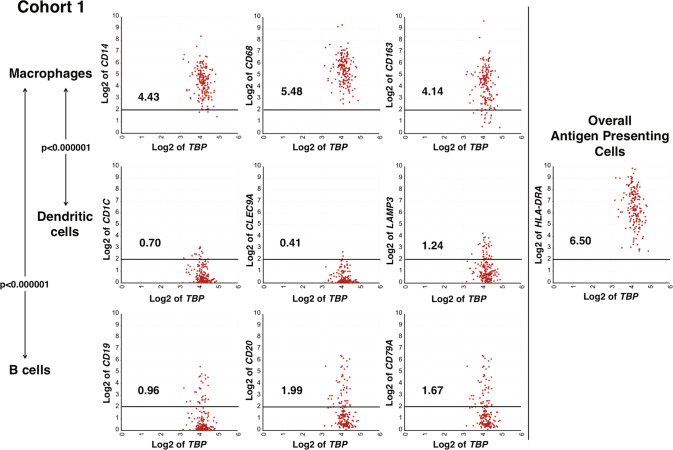
Macrophages are predominant classical HLA Class II positive cells in the TME of high-risk neuroblastoma. Macrophage signature genes (*CD14*, *CD68,* and *CD163*) were expressed at significantly higher levels compared to dendritic cell signature genes (*CD1C*, *CLEC9A,* and *LAMP3*) and B cell signature genes (*CD19*, *CD20,* and *CD79A*). All three genes in each cell type exhibited similar expression levels. The expression of *HLA-DRA* in high-risk neuroblastoma tissues was shown as a representative of the overall antigen-presenting cell population. Similar data were also obtained for Cohort 2 (see Fig. S2). To maximize accuracy of the analysis, we used three signature genes for a given cell type to evaluate relative abundance of each cell type. The expression unit of genes in Cohort 1 is Reads Per Million (RPM). Each number in the figure panels indicates the average expression level of the gene indicated. The horizontal bars at 2 Log2 RPM were included as a reference for the expression level. Statistical analysis was done using a two-tailed Student’s *t* test.

The relative abundance of TAMs, dendritic cells and B cells in high-risk neuroblastoma has not been well documented. To address this, we selected signature genes for dendritic cell and B cell through the procedure described in Supplemental Information and compare expression levels of these genes with levels of macrophage signature genes in high-risk neuroblastoma tissues. This analysis revealed that the expression levels of the dendritic cell marker genes and B cell signature genes were significantly lower than that of the macrophage marker genes (p < 0.000001 for both macrophages vs. B cells, and macrophages vs. dendritic cells) (Fig. 2 and S2). These data suggested that there was a low abundance of dendritic cells and B cells, and macrophages were the majority of the HLA-Class II positive cells in the TME of high-risk neuroblastoma tissues.

### Macrophage-derived *PD-L1* expression is associated with better outcome of high-risk neuroblastoma

**Fig. 3:**
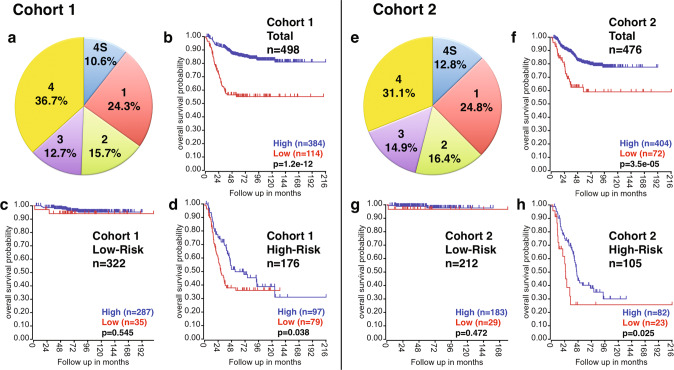
Prognostic significance of *PD-L1* expression in neuroblastoma. Two large neuroblastoma cohorts (Cohort 1: SEQC; Cohort 2: Kocak) were examined for the expression of *PD-L1* and its relationship to the patient survival. Distribution of disease stages of Cohort 1 and Cohort 2 demonstrates a similarity of the two cohorts in terms of the patients make up (**a** and **e**). For both Cohort 1 and Cohort 2, tumor stage was classified according to the International Neuroblastoma Staging System. Cohort 1 included the following tumors: stage 1 (*n* = 121, *MYCN*-amplified, *n* = 3), stage 2 (*n* = 78, *MYCN*-amplified, *n* = 5), stage 3 (*n* = 63, *MYCN*-amplified, *n* = 15), stage 4 (*n* = 183, *MYCN*-amplified, *n* = 65), and stage 4S (*n* = 53, *MYCN*-amplified, *n* = 4). Cohort 2 was composed of the following tumors: stage 1 (*n* = 153, *MYCN*-amplified, *n* = 5), stage 2 (*n* = 113, *MYCN*-amplified, *n* = 4), stage 3 (*n* = 91, *MYCN*-amplified, *n* = 15), stage 4 (*n* = 214, *MYCN*-amplified, *n* = 65), stage 4S (*n* = 78, *MYCN*-amplified, *n* = 4). The cutoff value for Cohort 1 was set at 2.044 Reads Per Million (RPM) for the total cohort and the high-risk subset, and 2.021 RPM for the low-risk subset (the closest value to 2.044 RPM among the low-risk subset). This allowed us to compare the effect of similar levels of PD-L1 positive cells on survival of neuroblastoma patients of different subsets. Note that a formal gene expression unit does not apply to Cohort 2 as the normalization of gene expression levels was done using the custom algorithm. The cutoff value for Cohort 2 was set at 301.2 for the total cohort and the low-risk subset, and 300.4 for the high-risk subset. The analysis was done using the R2 platform (http://r2.amc.nl).

We previously reported that macrophages were predominant PD-L1+ cells in neuroblastoma tissues at diagnosis based on multiplex IHC analysis [5]. In this report, we further extended this observation by examining the prognostic effect of *PD-L1* expression in two large neuroblastoma gene expression datasets, including SEQC-498 (Cohort 1) and Kocak-649 (Cohort 2) [30, 31] (Fig. 3). Survival analysis was performed on the total neuroblastoma cohorts, the low-risk and high-risk subsets. As shown in Fig. 3, high *PD-L1* expression was significantly associated with better outcome of the patients in the total cohorts (Fig. 3b, f) and the high-risk subsets (Fig. 3d, h) in both Cohorts 1 and 2. In contrast, no association was found between *PD-L1* expression and survival of patients of the low-risk subsets (Fig. 3c, g). This could be due to the fact that the overall immune activity in low-risk neuroblastoma is higher than that of the high-risk counterpart and that the macrophage-mediated anti-neuroblastoma effect could be secondary in low-risk neuroblastomas (Fig. S3).

### Overexpression of *PD-1* relative to *PD-L1* on TAMs is associated with poor prognosis of high-risk neuroblastoma

**Fig. 4:**
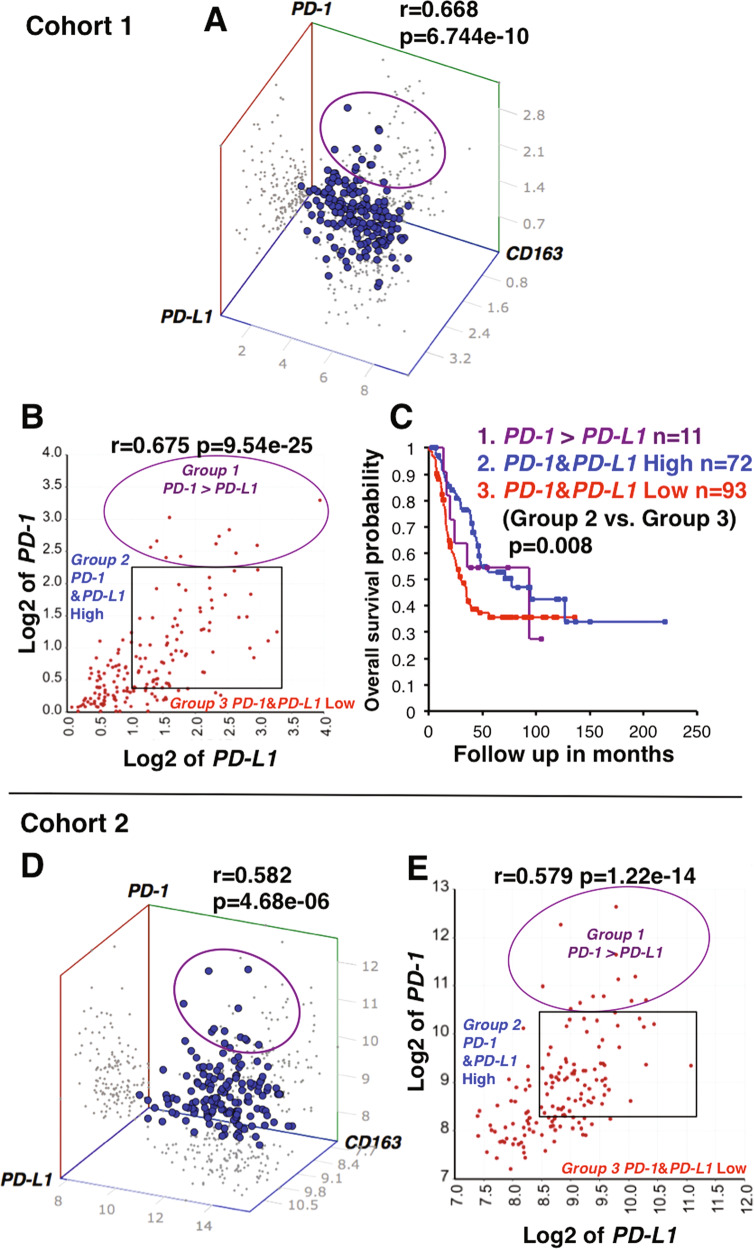
Co-expression of *PD-1* and *PD-L1* on M2-like macrophages and its prognostic implication for high-risk neuroblastoma. **A**, **D** 3D correlation analysis of *PD-1*, *PD-L1* and *CD163* expressions in high-risk neuroblastomas. A significant 3-way correlation among the expression of *PD-1*, *PD-L1* and *CD163* was observed in high-risk neuroblastomas of Cohorts 1 and 2. Notably, there was a population of *PD-L1* + tumors that overexpressed *PD-1* relative to *PD-L1* (indicated by the circle). **B**, **E** Relationship between *PD-1* and *PD-L1* expressions in high-risk neuroblastomas. *PD-1* expression was correlated significantly with *PD-L1* expression. However, there was a population of tumors in which *PD-1* expression levels exceeded *PD-L1* expression (indicated by the circle). Based on the expression levels of *PD-1* and *PD-L1*, the tumors can be divided into three groups: Group 1 exhibited high levels of both *PD-1* and *PD-L1* expression, but levels of *PD-1* expression were higher than those of *PD-L1* (*PD-1*/*PD-L1* > 1); Group 2 showed equally high levels of both *PD-1* and *PD-L1* expressions (*PD-1*/*PD-L1* ≈ 1); Group 3 showed low expression of both *PD-1* and *PD-L1*. **C** Prognostic implication of *PD-1* and *PD-L1* expressions in high-risk neuroblastomas of Cohort 1. Survival of patients in the Groups 1, 2, and 3 defined in Fig. 4B was compared. Group 2 with both *PD-1* and *PD-L1* high expression exhibited significantly better survival than Group 3 with both *PD-1* and *PD-L1* low expression. Group 1 showed a similar trend to Group 3. The expression unit of genes in Cohort 1 is Reads Per Million (RPM). Multivariable survival analysis (as shown in Fig. 4C for Cohort 1) could not be done on Cohort 2 because of the lack of necessary information in the R2. The custom algorithm was used to normalize the expression level of genes in Cohort 2, and therefore no formal unit applied to the expression level.

Previous studies suggest that PD-1/PD-L1 signaling in TAMs impairs their phagocytic capacity [17–19]. This prompted us to investigate the prognostic effect of *PD-1*/*PD-L1* co-expression on the survival of high-risk neuroblastoma patients. The 3D correlation analysis indicated that high-risk neuroblastomas co-expressed *PD-L1*, *PD-1*, and *CD163* (an M2 TAM marker) (Fig. 4A, D), suggesting the presence of M2-like TAMs with the PD-L1+PD-1+ phenotype. Moreover, three subsets of the tumors could also be present (Fig. 4B): Group 1 tumors overexpressed *PD-1* compared to *PD-L1* expression (*PD-1*/*PD-L1* > 1); Group 2 tumors expressed high levels of both *PD-1* and *PD-L1* (*PD-1*/*PD-L1* ≈ 1); Group 3 tumors expressed low levels of both *PD-1* and *PD-L1*. A similar distribution of high-risk tumors based on *PD-L1* and *PD-1* expressions was also observed in Cohort 2 (Fig. 4E). Survival analysis based on the combination of *PD-1* and *PD-L1* expressions (Fig. 4C) showed that there was a significant difference in survival between Group 2 (high *PD-1* and *PD-L1* with the ratio *PD-1*/*PD-L1* ≈ 1) and Group 3 (*PD-1* low and *PD-L1* low). In addition, Group 1 (*PD-1*/*PD-L1* > 1) exhibited poor survival, a similar trend to that of Group 3 (see Discussion).

### Possible status of TAMs in high-risk neuroblastoma tissues at diagnosis

Results of the survival analyses shown in Figs. 3,4C (Group 2 vs. Group 3 tumors) suggest an anti-tumor effect of macrophages in high-risk neuroblastoma tissues at diagnosis and therefore the presence of anti-tumor M1-like TAMs. However, most patients were deceased over time. This could be explained in part due to an increase in PD-L1+MDSC (myeloid-derived suppressor cells) [5] and progressive polarization of the pro-inflammatory/anti-tumor M1 TAMs to pro-tumor M2 TAMs in the TME. TAMs polarization is a biologically complex process, and macrophage phenotypes are much more diverse, which reveals many hybrid states forming a continuum of activation states [32, 33]. Therefore, M1/M2 markers cannot surely define the activation status of macrophages. These observations led us to evaluate the possible status of TAMs expressing CD68 (a pan-macrophage marker) and CD163 (an “M2” TAM marker) in high-risk neuroblastoma at diagnosis.

**Fig. 5:**
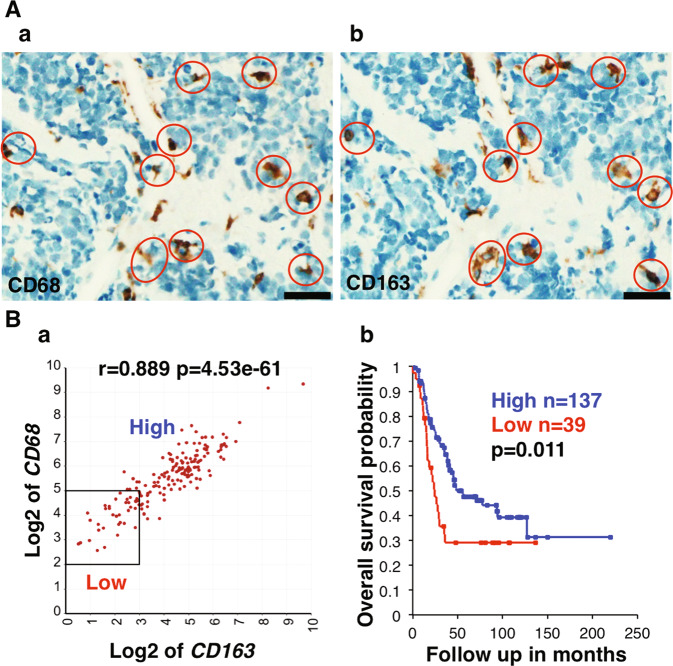
Prognostic implication of high-level expression of *CD68* and *CD163* in high-risk neuroblastoma. **A** The majority of tumor associated macrophages in unfavorable histology neuroblastoma coexpresses CD68 and CD163. Serial sections from an unfavorable histology neuroblastoma with *MYCN* amplification were stained with either (**a**) anti-CD68 or (**b**) anti-CD163 antibodies. Red circles indicate macrophages that co-express CD68 and CD163. The scale bar: 50μm. A similar observation was also made in a favorable histology neuroblastoma (see Fig. S4B). **B** (**a**) *CD68* expression was highly correlated with *CD163* expression in high-risk neuroblastomas of Cohort 1. (**b**) The high-risk neuroblastoma patients with high-level expressions of both *CD68* and *CD163* showed prolonged survival. A similar analysis could not be done for Cohort 2 due to the lack of necessary information embedded in the Kocak-649 dataset in the R2. The expression unit of genes in Cohort 1 is Reads Per Million (RPM).

IHC staining of high-risk neuroblastoma tissues with anti-CD68 and anti-CD163 antibodies revealed that a number of macrophages positive for these markers were present in a similar density and distribution pattern (Fig. S4A). Moreover, IHC staining of serial sections from an unfavorable histology *MYCN* amplified neuroblastoma demonstrated that the majority of CD68+ macrophages co-expressed CD163 (Fig. 5A). Survival analysis further showed that high-risk neuroblastoma patients from Cohort 1 having tumors with both high *CD68* and *CD163* expression exhibited better survival (Fig. 5B). This observation supports the hypothesis that most macrophages co-expressing high levels of *CD68* and *CD163* function as anti-tumor phagocytes in high-risk neuroblastomas at diagnosis.

### High-level co-expression of *SLAMF7* and *SH2D1B* is linked to the anti-high-risk neuroblastoma immunity

**Fig. 6:**
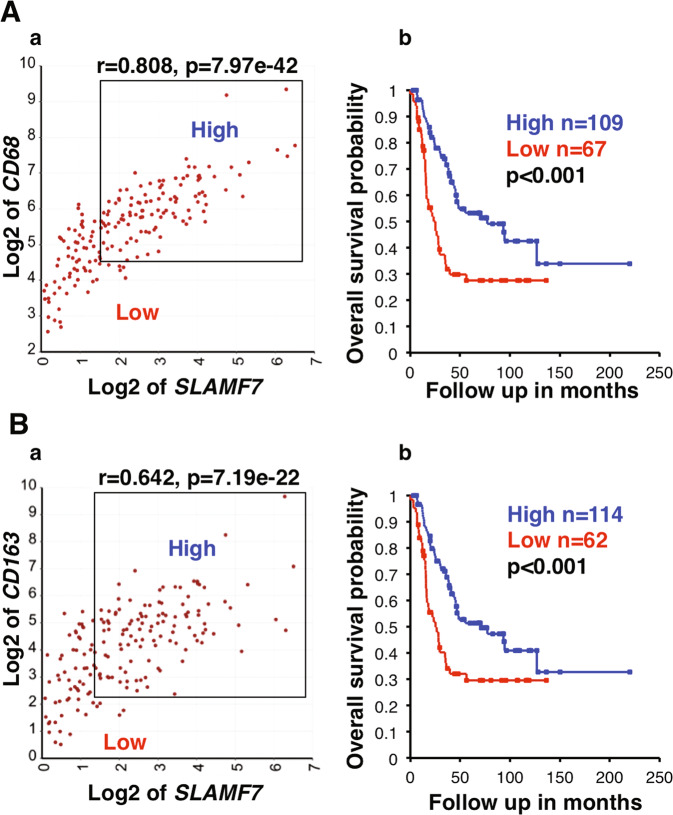
*SLAMF7* expression in macrophages is associated with prolonged survival of high-risk neuroblastoma patients. **A** (**a**) *CD68* and *SLAMF7* expressions were highly correlated with each other. (**b**) Combination of high *CD68* and high *SLAMF7* expressions was significantly associated with better survival of the high-risk neuroblastoma patients. **B** (**a**) *CD163* and *SLAMF7* expressions were highly correlated with each other. (**b**) Combination of high *CD163* and high *SLAMF7* expressions was significantly associated with better survival of the high-risk neuroblastoma patients. Cohort 1 was used in the analysis. The expression unit of genes in Cohort 1 is Reads Per Million (RPM).

Several molecular pathways activate macrophage phagocytosis, including Fcγ receptors, LRP1 and SLAMF7 pathways [34]. We have become interested in a potential involvement of the SLAMF7 pathway in tumor phagocytosis of macrophages in high-risk neuroblastoma because biological effect of the SLAMF7 pathway on the clinical behavior of neuroblastoma has not been addressed previously. The KEGG pathway analysis revealed that *SLAMF7* expression in high-risk neuroblastoma tissues of Cohort 1 (Table S1) and Cohort 2 (Table S2) was significantly associated with numerous immunological pathways, including those related to macrophage-functions, such as Osteoclast differentiation (*p* = 1.4e−22), Antigen processing and presentation (*p* = 2.0e−16), Lysosome (*p* = 4.2e−16) and Phagosome (p = 1.4e-15). Furthermore, high-level co-expression of *SLAMF7* and *CD68* was significantly associated with better outcome of high-risk neuroblastoma patients (Fig. 6Aa, b). As mentioned above, Fig. 5B data showed a similar pattern for co-expression of *CD68* and *CD163* and patients’ survival, suggesting M2-like TAMs could exhibit an anti-tumor effect. To confirm the above observations, we performed the additional survival analysis, which showed that high expression of both *SLAMF7* and *CD163* was indeed associated with longer survival of high-risk neuroblastoma (Fig. 6Ba, b).

**Fig. 7:**
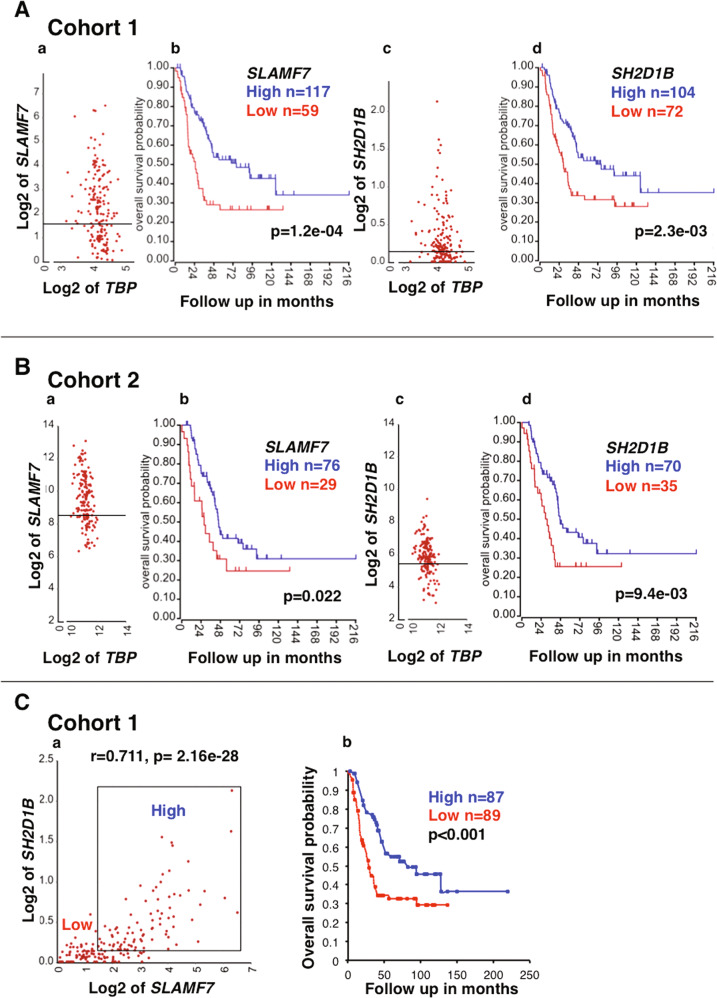
Potential involvement of the SLAMF7/EAT-2 pathway activation in macrophages in the anti-high-risk neuroblastoma immune response. **A** Cohort 1: (**a**) The expression of *SLAMF7*, encoding a macrophage activating receptor was detected in high-risk neuroblastomas of Cohort 1. (**b**) High *SLAMF7* expression was associated with better survival of high-risk neuroblastoma patients. Similar observations were made for *SH2D1B*, encoding the obligatory activating signal transduction molecule EAT-2 (**c**, **d**). The housekeeping gene *TBP* was used as a reference. The expression unit of genes in Cohort 1 is Reads Per Million (RPM). **B** Cohort 2: the SLAMF7/EAT-2 pathway in macrophages in high-risk neuroblastomas of Cohort 2. The expression of *SLAMF7* and *SH2D1B* was detected in high-risk neuroblastoma tissues of Cohort 2 (**a**, **c**). High expressions of both *SLAMF7* and *SH2D1B* were associated with better survival of high-risk neuroblastoma patients of Cohort 2 (**b**, **d**). The custom algorithm was used to normalize the expression level of genes in Cohort 2, and therefore no formal unit applied to the expression level. **C** (**a**) *SLAMF7* and *SH2D1B* expressions were highly correlated with each other. (**b**) Patients having high-risk neuroblastoma with high expression of both *SLAMF7* and *SH2D1B* exhibited better survival. Cohort 1 was used in the analysis. The expression unit of genes in Cohort 1 is Reads Per Million (RPM).

EAT-2 (encoded by *SH2D1B*) is the required adaptor for the SLAMF7 activation signal upon the homotypic interactions between SLAMF7 molecules. To address if the SLAMF7 pathway in macrophages could be activated and contribute to the anti-high-risk neuroblastoma immune response, we investigated the prognostic effect of *SLAMF7* and *SH2D1B*. We found that high expression of both *SLAMF7* and *SH2D1B* expression was significantly associated with better outcome of the patients in Cohort 1 (Fig. 7A**)** and Cohort 2 (Fig. 7B). Moreover, high-level co-expression of *SLAMF7* and *SH2D1B* was significantly associated with better survival of high-risk neuroblastoma patients (Fig. 7C).

**Fig. 8:**
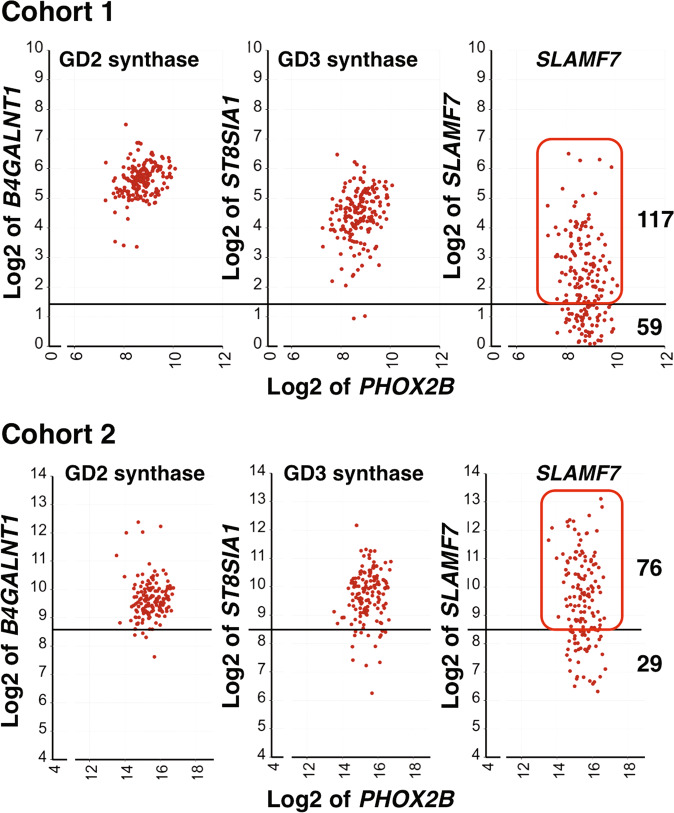
The expression of *SLAMF7* relative to those of *B4GALNT1* and *ST8SIA1* in high-risk neuroblastomas. Expression levels of *SLAMF7* in high-risk neuroblastomas of Cohort 1 and Cohort 2 were compared to those of neuroblastoma signature genes (*B4GALNT1* and *ST8SIA1*, encoding GD2 synthase and GD3 synthase, respectively). These enzymes are responsible for the expression of GD2 disialogangliosides on the neuroblastoma cell surface, providing an immunotherapy target. GD3 synthase is considered the rate-limiting enzyme of GD2 synthesis pathway and often subjected to epigenetic down-regulation. The neuroblastoma marker gene *PHOX2B* was used as a reference. The horizontal bars represent the cutoff values used for survival analysis of *SLAMF7* expression shown in Fig. 7A-a for Cohort 1 and Fig. 7B-a for Cohort 2. Based on the pattern of the *SLAMF7* expression, ~67% and 72% of high-risk neuroblastomas in Cohort 1 and Cohort 2, respectively, likely expressed *SLAMF7*. These patients showed better survival. The expression unit of genes in Cohort 1 is Reads Per Million (RPM). The custom algorithm was used to normalize the expression level of genes in Cohort 2, and therefore no formal unit applied to the expression level.

We next addressed if the trans-interaction between SLAMF7 on macrophages and neuroblastoma cells could occur by examining *SLAMF7* expression on the tumor cells relative to those of *B4GALNT1* (GD2 synthase gene) and *ST8SIA1* (GD3 synthase gene) in high-risk neuroblastoma tissues. *B4GALNT1* and *ST8SIA1* are responsible for the expression of GD2, the well-recognized target of neuroblastoma immunotherapy. As shown in Fig. 8, about 67% and 72% of the high-risk neuroblastoma patients in Cohort 1 and Cohort 2, respectively, who exhibited a better survival (Fig. 7A, B), likely expressed *SLAMF7*.

## Discussion

The significant progress in the immune checkpoint blockade therapy for adult cancers has brought a renewed interest in immunotherapy for pediatric malignancies, including neuroblastoma. This prompted investigators to examine the expression and prognostic effect of PD-L1 in neuroblastoma. Several investigators reported that neuroblastoma cells per se expressed PD-L1 [7–10]. Of which, three studies suggested that high PD-L1 expression was associated with poor survival of neuroblastoma patients [7, 8, 10]. In contrast, Aoki et al. described that neuroblastoma did not express PD-L1 [11]. Recently, Shirinbak et al. showed that the majority of neuroblastoma cells at diagnosis expressed little PD-L1, and its expression appeared up-regulated post-chemotherapy in a small population of neuroblastoma cells and that *PD-L1* expression was not associated with survival of neuroblastoma patients [12]. This study extended our earlier observation that neuroblastoma cells did not express PD-L1 whereas PD-L1 expression was detected on TAMs in the tumor tissues collected at diagnosis [5], and in fact, high *PD-L1* expression on macrophages was associated with better patients survival as a whole and of the high-risk neuroblastoma, but not the low-risk subset (Fig. 3).

An explanation for the discrepancy in the conclusions among the researchers on the prognostic effect of PD-L1 expression on neuroblastoma could be how PD-L1+ cells are identified. Precise detection of PD-L1+ cells in neuroblastoma tissues must be done by multiplex IHC assay, in which nuclear PHOX2B staining identifies neuroblastoma cells [5], and PD-L1+ cells are visualized by cell membrane staining by the corresponding antibody [5]. In addition, microscopic observation by clinical pathologists is required to avoid misidentification of dark brown pigments, hemosiderins as PD-L1+ cells. Another factor that could lead to the conflicting data is the size and the patients’ makeup of the study cohort. The cohorts used in this study well represent the overall neuroblastoma population (Fig. 3a, e). In addition, the two large neuroblastoma cohorts were used to analyze the prognostic effect of *PD-L1* expression, which minimized the possibility of drawing misrepresentative conclusions.

Our results suggest that there are *CD163*+ M2-like TAMs co-expressing *PD-L1* and *PD-1* in the TME of high-risk neuroblastomas (Fig. 4). In addition, the *PD-1*/*PD-L1* ratio could be considered a readout for the effector function of the macrophages and thereby patients’ survival. Patients bearing tumors with TAMs expressing high levels of *PD-1* and *PD-L1* as well as *PD-1*/*PD-L1* ratio ≈1 have a prolonged survival (Group 2, Fig. 4C). It remains to be proven that macrophages with this phenotype could phagocytose the tumor cells. We postulate that the balance between the inhibitory signal by PD-1/PD-L1 interaction and an activating signal such as the SLAMF7 pathway determines the overall activation status of macrophages. When the *PD-1*/*PD-L1* ratio >1, the patients do poorly (Group 1, Fig. 4C). This may involve exhaustion of macrophages and a decrease in their phagocytic potency as previously described [17]. Therefore, PD-1 blockade therapy could show an efficacy against high-risk neuroblastomas with the *PD-1/PD-L1* ratio >1 phenotype. The low expression of both *PD-L1* and *PD-1* in Group 3 (Fig. 4B, E) suggests poor tumor-infiltrating macrophages in the TME of these high-risk neuroblastomas. In addition, most macrophages co-expressing high levels of *CD68* and *CD163* could function as anti-tumor phagocytes in high-risk neuroblastomas at diagnosis (Fig. 5). It remains to be seen whether this *CD68*+*CD163*+ macrophages population is the same as those of the Group 2 tumors (*CD163*+ and *PD-1*/*PD-L1* ratio ≈ 1).

Blockade of the PD-1/PD-L1 pathway has been shown to enhance phagocytic function of macrophages in adult cancers [17–19] and lengthen survival in mouse models of cancer in a macrophage-dependent fashion [17]. However, early clinical trials have reported that anti-PD-1 or anti-PD-L1 antibody alone had little efficacy in pediatric cancers including neuroblastoma [35–37]. Nonetheless, these clinical trials were conducted on the patients who were heavily pre-treated with chemotherapy, which could have adversely affected the immune competency of the patient [6]. In addition, based on our data, PD-1 blockade therapy may only be efficacious for the high-risk neuroblastomas with the *PD-1/PD-L1* ratio >1 phenotype (Group 1, Fig. 4B, E). Furthermore, macrophages have been shown to uptake anti-PD-1 antibodies through their Fcγ receptors, thereby limiting efficacy in animal models [38]. To prevent this, a high affinity PD-1 variant that functions as a PD-L1 blocker is developed and tested for its efficacy in mouse model [17, 39].

Macrophages can play an anti-tumor or a pro-tumor function in cancers, and our data reiterate the complicated topic on polarization of TAMs. Generally, expression of the M2 marker CD163 is associated with tumor aggressiveness and shorter overall survival in many cancers [40, 41]. However, the recent report suggests that an increased density of CD68+CD163+ macrophages in tumor tissues is in fact associated with up-regulated immune signaling and improved survival of patients with gastric cancer [42]. Indeed, our data suggest that CD163+ macrophages could function as anti-tumor phagocytes in high-risk neuroblastoma at diagnosis. This issue has become even more intriguing when various approaches have been conducted to induce the full phagocytic capacity of TAMs by reprogramming pro-tumor to anti-tumor TAMs in adult cancers [43–45]. However, these therapeutic strategies showed limited efficacy in human clinical trials against solid tumors [43, 45]. Drug-induced conversions of M2 to M1 TAMs in pre-clinical studies in neuroblastomas have also been reported, which shows inhibition of tumor growth [46]. It remains to be seen if those experimental therapeutics would have efficacy against high-risk neuroblastoma in human clinical trials.

Our data suggest that the majority of high-risk neuroblastomas could express *SLAMF7* (Fig. 8). Homotypic interaction could then occur between the *SLAMF7*+ tumor cells and macrophages, leading to macrophage activation and tumor phagocytosis. On the other hand, SLAMF7 negative tumor cells could also be killed by the bystander effect of the activated macrophages nearby when agonistic anti-SLAMF7 antibodies were administered. In the presence of anti-SLAMF7 antibodies, the SLAMF7+ tumor cells could also be killed by ADCC. In fact, an FDA-approved humanized anti-SLAMF7 monoclonal antibody elotuzumab [47, 48] is available for clinical testing. A combination of PD-1 blockade and anti-SLAMF7 could therefore maximize the effector function of macrophages in a subpopulation of high-risk neuroblastomas (Group 1, Fig. 4C). We have reported CD4 CTL as an important effector of high-risk neuroblastoma [5], and work by others on adult cancers has indicated that cytotoxic activity of tumor-specific cytolytic CD4 T cells is in part dependent on SLAMF7 [49]. Thus, the use of anti-SLAMF7 antibody as therapeutics for high-risk neuroblastoma could enhance effector functions of both CD4 CTL and macrophages.

The SLAMF7 pathway is our focus in this study. Nonetheless, our preliminary analysis indicates that other SLAM family genes are expressed at compatible levels to that of *SLAMF7* in high-risk neuroblastoma tissues (*SLAMF2*, *SLAMF5,* and *SLAMF8*). In contrast, high-risk neuroblastoma expressed low levels of *SLAMF3* and *SLAMF4*, which have been reported as “do not eat me signals” in hematopoietic malignancies [50]. In addition, among Fcγ receptor genes, *FCGR2A* and *FCGR3A*, encoding activating receptors, are expressed at relatively high levels in high-risk neuroblastoma tissues, suggesting the potential roles of these FCGRs in macrophage-mediated anti-high-risk neuroblastoma immunity. *LRP1* is also expressed at high levels in high-risk neuroblastoma tissues. Thus, it would be of interest to explore how the interplay among these pathways affects the activation status of macrophages in high-risk neuroblastoma, which may lead to new and innovative therapeutic interventions for high-risk neuroblastoma in future studies.

In summary, neuroblastoma cells evade CD8+CTL-mediated immunity due to their lack of HLA Class I expression and low mutational burden. To overcome this hurdle, the immune system engages alternative effector cells (macrophages and CD4 CTL [5]) to eliminate high-risk neuroblastoma cells. Various immunotherapy protocols could be designed to maximize tumor-killing capacity of these effector cells [5], and the availability of humanized antibodies for adult cancers would help accelerate progress for immunotherapy against high-risk neuroblastoma. Apparently, there are patients having “extremely cold” tumors with poor tumor-infiltrating macrophages and CD4 CTL (this study and [5]). For these patients, an alternative therapeutic approach would be needed. Neuroblastoma often metastasizes to the bone marrow where hematopoiesis occurs. Consequently, such metastases could suppress the development of functional immune cells. Therefore, controlling bone marrow metastases and protecting hematopoietic stem cells are essential to a successful immunotherapy for high-risk neuroblastoma. This idea is supported by the observation that bone marrow metastases of breast cancer cells adversely affect the immune activity of the host [51]. Finally, this study has provided a framework for pursuing a high content analysis on a large high-risk neuroblastomas cohort to definitely define the immune cell landscape of this disease at single cell levels.

## Materials and methods

### The study cohort

Two cohorts including neuroblastoma specimens collected at diagnosis were used in this study. Cohort 1 was composed of 498 tumors, of which 322 were low-risk tumors and 176 were high-risk cases [30]. The high- and low-risk categories of Cohort 1 were determined by the original study [30], and the high-risk subset included tumors of stage 4 disease, >18 months at diagnosis and patients of any age and stage with *MYCN*-amplified tumors, whereas the rest are the low-risk tumors.

Cohort 2 included 649 samples, of which 476 cases had survival data [31]. The low- and high-risk subsets of Cohort 2 were defined by this study: the low-risk subset (*n* = 212) included cases less than 18-month old at diagnosis with stage 1, 2, and 4S diseases and having no *MYCN* amplification; the high-risk subset included cases over 18-month old and with stage 4 disease (*n* = 148, of which 105 cases with survival data).

### Gene expression analysis

We employed the two primary neuroblastoma gene expression datasets: SEQC-498 (GEO ID: gse62564) [30] using an RNA-seq methodology and Kocak-649 (GEO ID: gse45547) [31] using a microarray methodology. The datasets have been validated by the previous publications [30, 31]. The gene expression analysis was performed by the R2 platform (http://r2.amc.nl). We also employed other resources: the 3D plotting program by Doka (https://www.doka.ch/Excel3Dscatterplot.htm) and the multiple correlation analysis by Zaiontz (https://www.real-statistics.com/correlation/multiple-correlation/). KEGG pathway analysis was performed by first identifying genes whose expressions were significantly correlated with the expression of a gene of interest. The resultant gene set was run against the KEGG pathway gene sets using the R2 with the *p* value cutoff of 0.01. The statistical analysis was done by the algorithms embedded in the R2 platform (https://hgserver1.amc.nl/r2/help/r2_tutorials.pdf).

### Multi-variable survival analysis

The multi-variable survival analysis was done using the software written by Lucijanić [52]. The statistical analysis was performed by the algorithm embedded in the software. *p* < 0.05 was considered statistically significant. Due to the information on OS-bin and OS-time of Cohort 2 were hidden in the R2 platform, we could not perform multiple gene expression survival analyses for Cohort 2.

### Immunohistochemistry assay

Diagnostic neuroblastoma samples from the Children’s Oncology Group (COG) and Stanford University, School of Medicine, Department of Pathology were used. After pathology review according to the protocol of the COG Neuroblastoma Biology Study, unused sections were available for immunostaining. Those cases were filed at the COG Neuroblastoma Pathology Reference Laboratory, which were identified only by the COG accession number and not associated with patient information. IRB approval of the cases was obtained at the time of study enrollment by the contributing institution. Neuroblastoma specimens archived at Stanford University were used to perform IHC staining on serial sections of the tumor tissues. Formalin-fixed paraffin embedded tumor sections were subjected to IHC analysis. Clone KP1 for CD68 (Dako) and clone MRQ-26 for CD163 (Ventana/Cell Marque) were used to stain the corresponding antigens. The automated IHC processor was used to process tumor sections according to the manufacturer’s instructions (Ventana Ultra, Roche). Immunohistochemical evaluation was performed in the representative and viable tumor areas away from necrosis and fibrosis.

## Supplementary information

Supplementary Information

Supplementary Figures

Supplementary Table S1

Supplementary Table S2

## Data Availability

Publicly available datasets were analyzed in this study. These data can be found in GEO: GSE62564; GSE45547.

## References

[1] Irwin MS, Naranjo A, Zhang FF, Cohn SL, London WB, Gastier-Foster JM, et al. Revised neuroblastoma risk classification system: a report from the Children’s Oncology Group. J Clin Oncol. 2021;39:3229–41. 10.1200/JCO.21.00278 PMC8500606 34319759

[2] Whelan JP, Chatten J, Lampson LA. HLA class I and b2-microglobulin expression in frozen and formaldehyde-fixed paraffin sections of neuroblastoma tumors. Cancer Res. 1985;45:5976–83. 3902214

[3] Wölfl M, Jungbluth AA, Garrido F, Cabrera T, Meyen-SouthardS, Spitz R, et al. Expression of MHC class I, MHC class II, and cancer germlineantigens in neuroblastoma. Cancer Immunology, Immunotherapy. 2005;54:400–6 10.1007/s00262-004-0603-z PMC11034322 15449039

[4] Lawrence MS, Stojanov P, Polak P, Kryukov GV, Cibulskis K, Sivachenko A, et al. Mutational heterogeneity in cancer and the search for newcancer-associated genes. Nature. 2013;499:214. 10.1038/nature12213 PMC3919509 23770567

[5] Tang XX, Shimada H, Ikegaki N (2021). Clinical relevance of CD4 cytotoxic T cells in high-risk neuroblastoma. Front Immunol.

[6] Chakraborty N, Bilgrami S, Maness L, Guo C, Perez-Diez A, Mukherji B (1999). Myeloablative chemotherapy with autologous peripheral blood stem cell transplantation for metastatic breast cancer: immunologic consequences affecting clinical outcome. Bone Marrow Transpl.

[7] Melaiu O, Mina M, Chierici M, Boldrini R, Jurman G, Romania P (2017). PD-L1 is a therapeutic target of the bromodomain inhibitor JQ1 and, combined with HLA Class I, a promising prognostic biomarker in neuroblastoma. Clin Cancer Res.

[8] Majzner RG, Simon JS, Grosso JF, Martinez D, Pawel BR, Santi M (2017). Assessment of programmed death-ligand 1 expression and tumor-associated immune cells in pediatric cancer tissues. Cancer.

[9] Silva MA, Triltsch N, Leis S, Kanchev I, Tan TH, Van Peel B (2020). Biomarker recommendation for PD-1/PD-L1 immunotherapy development in pediatric cancer based on digital image analysis of PD-L1 and immune cells. J Pathol Clin Res.

[10] Zuo S, Sho M, Sawai T, Kanehiro H, Maeda K, Yoshida M (2020). Potential role of the PD-L1 expression and tumor-infiltrating lymphocytes on neuroblastoma. Pediatr Surg Int.

[11] Aoki T, Hino M, Koh K, Kyushiki M, Kishimoto H, Arakawa Y (2016). Low frequency of programmed death Ligand 1 expression in pediatric cancers. Pediatr Blood Cancer.

[12] Shirinbak S, Chan RY, Shahani S, Muthugounder S, Kennedy R, Hung LT (2021). Combined immune checkpoint blockade increases CD8+CD28+PD-1+ effector T cell and provides a therapeutic strategy for patients with neuroblastoma. OncoImmunology.

[13] Dong H, Zhu G, Tamada K, Chen L (1999). B7-H1, a third member of the B7 family, co-stimulates T-cell proliferation and interleukin-10 secretion. Nat Med.

[14] Sharpe AH, Wherry EJ, Ahmed R, Freeman GJ (2007). The function of programmed cell death 1 and its ligands in regulating autoimmunity and infection. Nat Immunol.

[15] Patel SP, Kurzrock R (2015). PD-L1 expression as a predictive biomarker in cancer immunotherapy. Mol Cancer Therapeutics.

[16] Okazaki T, Honjo T (2007). PD-1 and PD-1 ligands: from discovery to clinical application. Int Immunol.

[17] Gordon SR, Maute RL, Dulken BW, Hutter G, George BM, McCracken MN (2017). PD-1 expression by tumour-associated macrophages inhibits phagocytosis and tumour immunity. Nature.

[18] Hartley GP, Chow L, Ammons DT, Wheat WH, Dow SW (2018). Programmed cell death Ligand 1 (PD-L1) signaling regulates macrophage proliferation and activation. Cancer Immunol Res.

[19] Strauss L, Mahmoud MAA, Weaver JD, Tijaro-Ovalle NM, Christofides A, Wang Q (2020). Targeted deletion of PD-1 in myeloid cells induces antitumor immunity. Sci Immunol.

[20] Cannons JL, Tangye SG, Schwartzberg PL (2011). SLAM family receptors and SAP adaptors in immunity. Annu Rev Immunol.

[21] O’Connell P, Pepelyayeva Y, Blake MK, Hyslop S, Crawford RB, Rizzo MD (2019). SLAMF7 is a critical negative regulator of IFN-α-mediated CXCL10 production in chronic HIV infection. J Immunol.

[22] Morra M, Lu J, Poy F, Martin M, Sayos J, Calpe S (2001). Structural basis for the interaction of the free SH2 domain EAT-2 with SLAM receptors in hematopoietic cells. EMBO J.

[23] Cruz-Munoz ME, Dong Z, Shi X, Zhang S, Veillette A (2009). Influence of CRACC, a SLAM family receptor coupled to the adaptor EAT-2, on natural killer cell function. Nat Immunol.

[24] Pérez-Quintero LA, Roncagalli R, Guo H, Latour S, Davidson D, Veillette A (2014). EAT-2, a SAP-like adaptor, controls NK cell activation through phospholipase Cγ, Ca++, and Erk, leading to granule polarization. J Exp Med.

[25] Reid GS, Shan X, Coughlin CM, Lassoued W, Pawel BR, Wexler LH (2009). Interferon-gamma-dependent infiltration of human T cells into neuroblastoma tumors in vivo. Clin Cancer Res.

[26] Carlson LM, Rasmuson A, Idborg H, Segerström L, Jakobsson PJ, Sveinbjörnsson B (2013). Low-dose aspirin delays an inflammatory tumor progression in vivo in a transgenic mouse model of neuroblastoma. Carcinogenesis.

[27] Berbegall AP, Villamón E, Tadeo I, Martinsson T, Cañete A, Castel V (2014). Neuroblastoma after childhood: prognostic relevance of segmental chromosome aberrations, ATRX protein status, and immune cell infiltration. Neoplasia.

[28] Wu HW, Sheard MA, Malvar J, Fernandez GE, DeClerck YA, Blavier L (2019). Anti-CD105 antibody eliminates tumor microenvironment cells and enhances Anti-GD2 antibody immunotherapy of neuroblastoma with activated natural killer cells. Clin Cancer Res.

[29] Zhang P, Wu X, Basu M, Dong C, Zheng P, Liu Y (2017). MYCN amplification is associated with repressed cellular immunity in neuroblastoma: an in silico immunological analysis of TARGET database. Front Immunol.

[30] Zhang W, Yu Y, Hertwig F, Thierry-Mieg J, Zhang W, Thierry-Mieg D (2015). Comparison of RNA-seq and microarray-based models for clinical endpoint prediction. Genome Biol.

[31] Kocak H, Ackermann S, Hero B, Kahlert Y, Oberthuer A, Juraeva D (2013). Hox-C9 activates the intrinsic pathway of apoptosis and is associated with spontaneous regression in neuroblastoma. Cell Death Dis.

[32] DeNardo DG, Ruffell B (2019). Macrophages as regulators of tumour immunity and immunotherapy. Nat Rev Immunol.

[33] Mehta AK, Kadel S, Townsend MG, Oliwa M, Guerriero JL (2021). Macrophage biology and mechanisms of immune suppression in breast cancer. Front Immunol.

[34] Feng M, Jiang W, Kim BYS, Zhang CC, Fu YX, Weissman IL (2019). Phagocytosis checkpoints as new targets for cancer immunotherapy. Nat Rev Cancer.

[35] Davis KL, Fox E, Merchant MS, Reid JM, Kudgus RA, Liu X (2020). Nivolumab in children and young adults with relapsed or refractory solid tumours or lymphoma (ADVL1412): a multicentre, open-label, single-arm, phase 1-2 trial. Lancet Oncol.

[36] Geoerger B, Zwaan CM, Marshall LV, Michon J, Bourdeaut F, Casanova M (2020). Atezolizumab for children and young adults with previously treated solid tumours, non-Hodgkin lymphoma, and Hodgkin lymphoma (iMATRIX): a multicentre phase 1-2 study. Lancet Oncol.

[37] Geoerger B, Kang HJ, Yalon-Oren M, Marshall LV, Vezina C, Pappo A (2020). Pembrolizumab in paediatric patients with advanced melanoma or a PD-L1-positive, advanced, relapsed, or refractory solid tumour or lymphoma (KEYNOTE-051): interim analysis of an open-label, single-arm, phase 1–2 trial. Lancet Oncol.

[38] Arlauckas SP, Garris CS, Kohler RH, Kitaoka M, Cuccarese MF, Yang KS, et al. In vivo imaging reveals a tumor-associated macrophage-mediated resistance pathway in anti-PD-1 therapy. Sci Transl Med. 2017;9:eaal3604. 10.1126/scitranslmed.aal3604 PMC5734617 28490665

[39] Maute RL, Gordon SR, Mayer AT, McCracken MN, Natarajan A, Ring NG (2015). Engineering high-affinity PD-1 variants for optimized immunotherapy and immuno-PET imaging. Proc Natl Acad Sci USA.

[40] Shiraishi D, Fujiwara Y, Horlad H, Saito Y, Iriki T, Tsuboki J (2018). CD163 is required for protumoral activation of macrophages in human and murine sarcoma. Cancer Res.

[41] Hu JM, Liu K, Liu JH, Jiang XL, Wang XL, Chen YZ (2017). CD163 as a marker of M2 macrophage, contribute to predicte aggressiveness and prognosis of Kazakh esophageal squamous cell carcinoma. Oncotarget.

[42] Huang YK, Wang M, Sun Y, Di Costanzo N, Mitchell C, Achuthan A (2019). Macrophage spatial heterogeneity in gastric cancer defined by multiplex immunohistochemistry. Nat Commun.

[43] Anderson NR, Minutolo NG, Gill S, Klichinsky M (2021). Macrophage-based approaches for cancer immunotherapy. Cancer Res.

[44] Jahchan NS, Mujal AM, Pollack JL, Binnewies M, Sriram V, Reyno L (2019). Tuning the tumor myeloid microenvironment to fight cancer. Front Immunol.

[45] Cai H, Zhang Y, Wang J, Gu J (2021). Defects in macrophage reprogramming in cancer therapy: the negative impact of PD-L1/PD-1. Front Immunol.

[46] Liu KX, Joshi S (2020). “Re-educating” tumor associated macrophages as a novel immunotherapy strategy for neuroblastoma. Front Immunol.

[47] Hsi ED, Steinle R, Balasa B, Szmania S, Draksharapu A, Shum BP (2008). CS1, a potential new therapeutic antibody target for the treatment of multiple myeloma. Clin Cancer Res.

[48] Tai YT, Dillon M, Song W, Leiba M, Li XF, Burger P (2008). Anti-CS1 humanized monoclonal antibody HuLuc63 inhibits myeloma cell adhesion and induces antibody-dependent cellular cytotoxicity in the bone marrow milieu. Blood.

[49] Cachot A, Bilous M, Liu YC, Li X, Saillard M, Cenerenti M, et al. Tumor-specific cytolytic CD4 T cells mediate immunity against human cancer. Sci Adv. 2021;7:eabe3348. 10.1126/sciadv.abe3348 PMC7909889 33637530

[50] Li D, Xiong W, Wang Y, Feng J, He Y, Du J (2022). SLAMF3 and SLAMF4 are immune checkpoints that constrain macrophage phagocytosis of hematopoietic tumors. Sci Immunol.

[51] Monteran L, Ershaid N, Sabah I, Fahoum I, Zait Y, Shani O (2020). Bone metastasis is associated with acquisition of mesenchymal phenotype and immune suppression in a model of spontaneous breast cancer metastasis. Sci Rep.

[52] Lucijanić M (2016). Survival analysis in clinical practice: analyze your own data using an Excel workbook. Croat Med J.

