# Supplementary material for: Macrophage-mediated anti-tumor immunity against high-risk neuroblastoma
Source: Genes Immun. 2022 May 7;23(3-4):129–40. doi: 10.1038/s41435-022-00172-w (PMC9232393; doi:10.1038/s41435-022-00172-w)
Supplement: Supplementary file 1 — Supplementary Information [file 41435_2022_172_MOESM1_ESM.pdf]

Supplementary Information

Determination of dendritic cell and B cell marker genes used in gene expression analysis for high-risk neuroblastoma

We first determined the most appropriate dendritic cell signature genes for gene expression profiling in high-risk neuroblastoma. According to the recent scRNA-seq study on tumor infiltrating myeloid cells by Chen et al. (1), *CD1C*, *CLEC9A* and *LAMP3* are signature genes of cDC1 (common dendritic cell-1), cDC2 (common dendritic cell-2) and tumor infiltrating DC, respectively. Based on this information, we examined the expression of *CD1C*, *CLEC9A* and *LAMP3* in high-risk neuroblastomas of Cohort 1 (SEQC). The expression of these genes was highly correlated to each other (*CD1C:CLEC9A*, r=0.820 p=4.83e-44), (*CD1C:LAMP3*, r=0.803 p=6.90e-41) and (*CLEC9A:LAMP3*, r=0.839 p=8.41e-48) in high-risk neuroblastoma of Cohort 1, suggesting that they could represent the overall dendritic cell population in high-risk neuroblastoma tissues. In addition, the expression of these genes is restricted to dendritic cells in normal tissues and cell types (Human Protein Atlas: https://www.proteinatlas.org/). Subsequently, we used *CD1C*, *CLEC9A* and *LAMP3* as dendritic cell signature genes to evaluate relative abundance of dendritic cells in high-risk neuroblastoma tissues (see **Figure 2** for Cohort 1). Note that *CLEC9A* was replaced with *TLR8* for Cohort 2 because the Cohort 2 gene expression platform did not include *CLEC9A* probes (see **Figure S2** for Cohort 2).

We also determined B cell signature genes for gene expression profiling analysis of high-risk neuroblastoma. Based on previous scRNA seq studies focused on tumor-infiltrating B cells in human solid tumors (2, 3), we selected several candidate signature genes for B cells, including *BLK*, *CD19*, *CD20*, *CD79A*, *CLEC17A*, *FCRL2, FCRLA, PAX5*, *TNFRSF13B* and *TLR10*. Some of these genes could also be expressed in non-B cells. Therefore, the expression of the above genes in normal tissues and cell types was assessed using Human Protein Atlas (https://www.proteinatlas.org/). This analysis showed that the expression of *CD19*, *CD20*, *CD79A*, *CLEC17A* and *PAX5* was more restricted to B cell lineage. Correlation analysis using

high-risk neuroblastomas of Cohort 1 (SEQC) revealed that the expression of *CD79A* that encodes a component of the B cell receptor complex was significantly correlated with that of *CD19* (r=0.947, p=9.98e-318), *CD20/MS4A1* (r=0.96, p=3.26e-273), *CLEC17A* (r=0.9.18, p=3.92e-198) and *PAX5* (r=0.854, p=1.63e-140). Based on this analysis, we chose *CD19*, *CD20* and *CD79A* as B cell signature genes to evaluate relative abundance of B cells in high-risk neuroblastoma tissues (**Figure 2** and **Figure S2**).

1. Cheng S, Li, Z, Gao, R, Xing, B, Gao, Y, Yang, Y, et al. A pan-cancer single-cell transcriptional atlas of tumor infiltrating myeloid cells. *Cell* (2021) 184 (3):792-809.e23. doi:10.1016/j.cell.2021.01.010

2. Chen J, Tan, Y, Sun, F, Hou, L, Zhang, C, Ge, T, et al. Single-cell transcriptome and antigen-immunoglobin analysis reveals the diversity of B cells in non-small cell lung cancer. *Genome Biol* (2020) 21 (1):152. doi:10.1186/s13059-020-02064-6

3. Hladíková K, Koucký, V, Bouček, J, Laco, J, Grega, M, Hodek, M, et al. Tumor-infiltrating B cells affect the progression of oropharyngeal squamous cell carcinoma via cell-to-cell interactions with CD8(+) T cells. *J Immunother Cancer* (2019) 7 (1):261. doi:10.1186/s40425-019-0726-6
