# Supplementary material for: Macrophage-mediated anti-tumor immunity against high-risk neuroblastoma
Source: Genes Immun. 2022 May 7;23(3-4):129–40. doi: 10.1038/s41435-022-00172-w (PMC9232393; doi:10.1038/s41435-022-00172-w)
Supplement: Supplementary file 2 — Supplementary Figures [file 41435_2022_172_MOESM2_ESM.pdf]

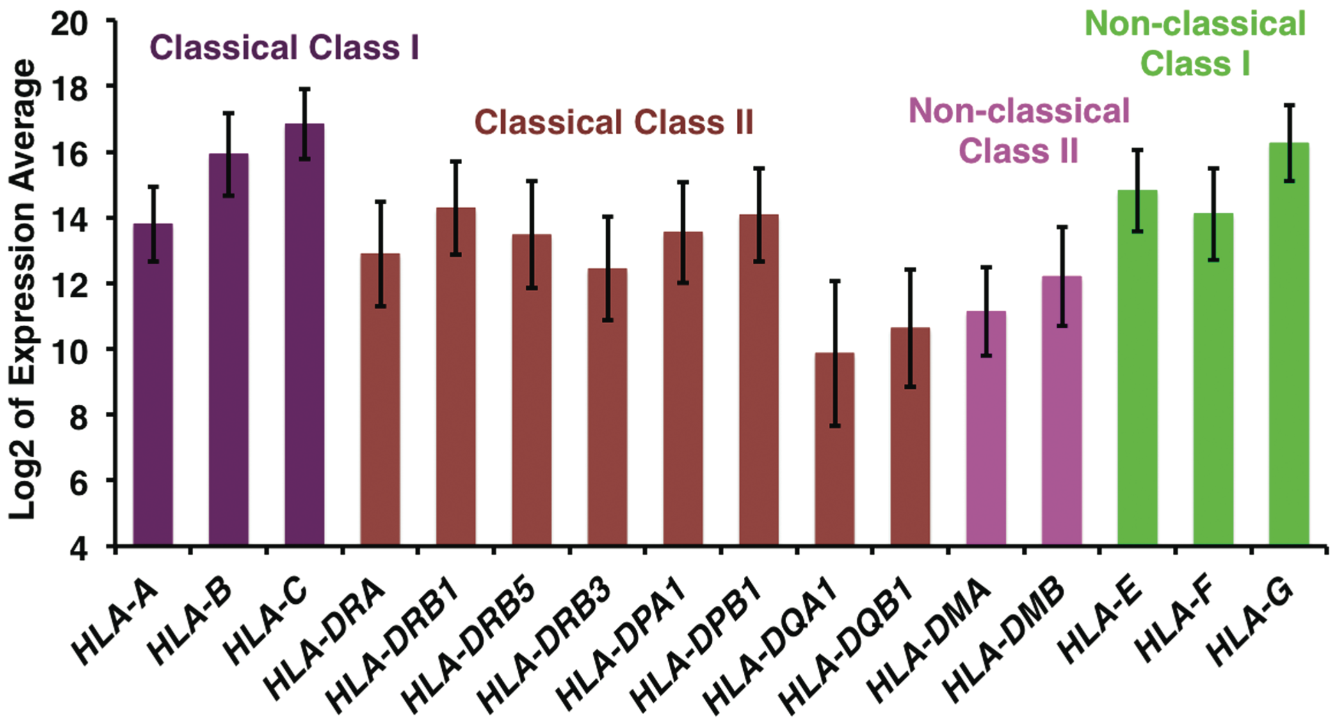

Fig. S1. High-level expression of classical HLA Class II genes in high-risk neuroblastomas of Cohort 2. HLA-related gene expression was examined for the high-risk subset of Cohort 2 by the R2. High-level expression of classical HLA-Class II genes (*HLA-DRA, HLA-DRB1, HLA-DRB3, HLA-DRB5, HLA-DPA1*, and *HLA-DPB1*) was detected in high-risk neuroblastoma tissues.

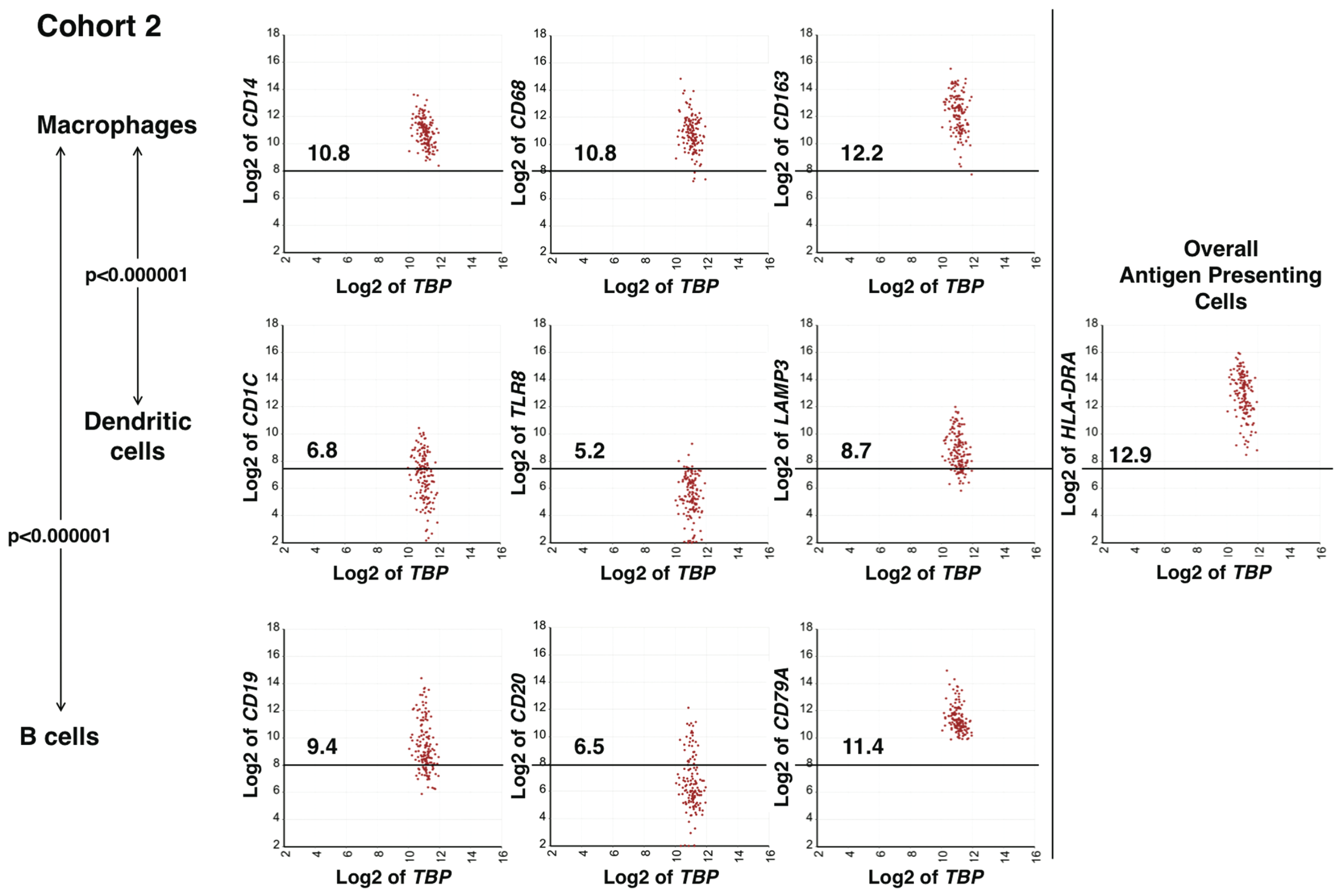

Fig. S2. Macrophages are predominant classical HLA Class II positive cells in the TME of high-risk neuroblastomas of Cohort 2. Macrophage signature genes (*CD14*, *CD68* and *CD163*) were expressed at significantly higher levels compared to dendritic cell signature genes (*CD1C*, *TLR8* and *LAMP3*) and B cell signature genes (*CD19*, *CD20* and *CD79A*). Note that *CLEC9A* that was used as a dendritic cell signature gene in Cohort 1 was replaced with *TLR8* for Cohort 2 because the Cohort 2 gene expression platform did not include *CLEC9A* probes. All three genes in each cell type exhibited very similar expression levels. Statistical analysis was done using a two-tailed Student's t-test.

Cohort 1

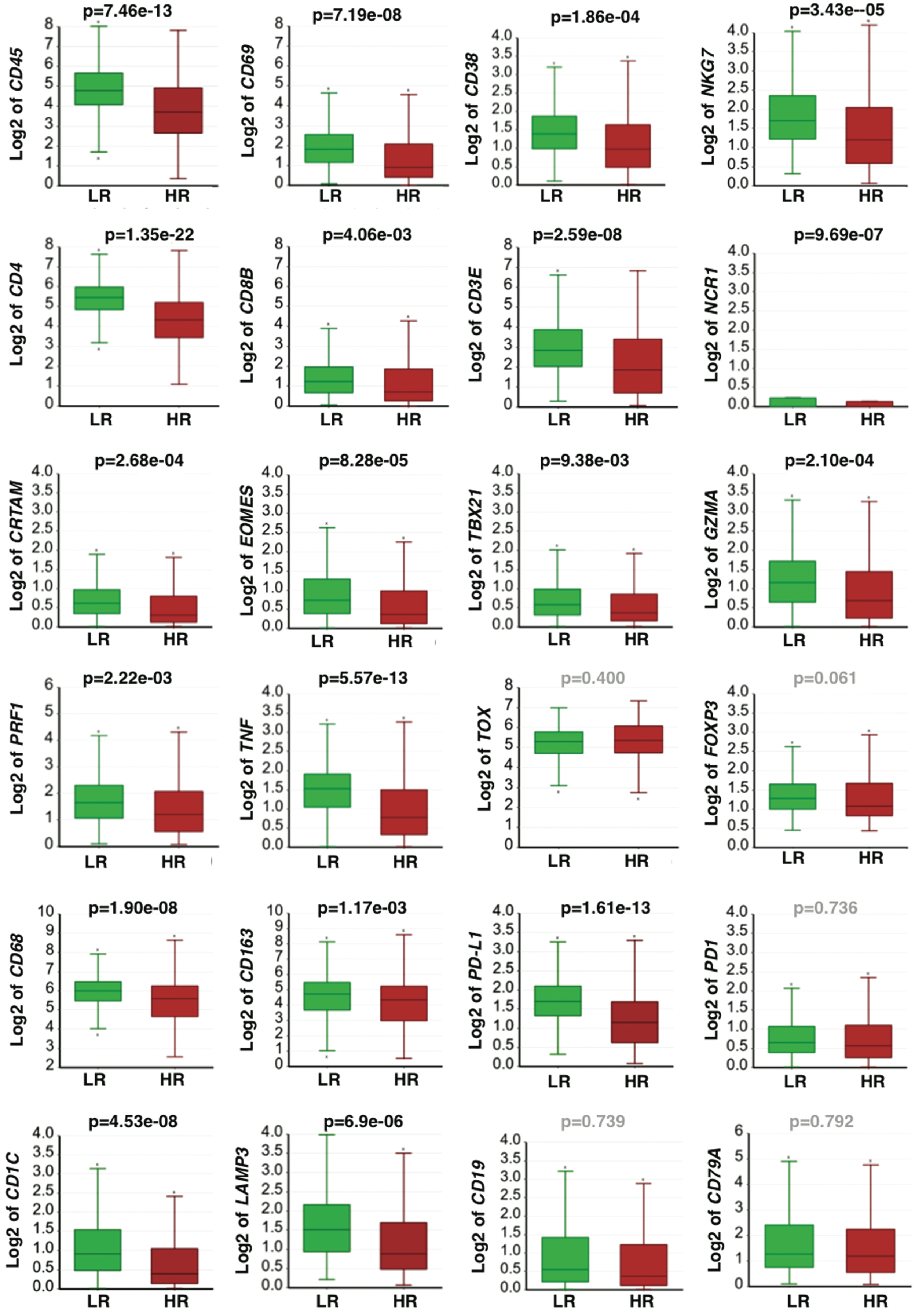
p=7.46e-13
Log2 of CD45
p=7.19e-08
Log2 of CD69
p=1.86e-04
Log2 of CD38
p=3.43e--05
Log2 of NKG7
p=1.35e-22
Log2 of CD4
p=4.06e-03
Log2 of CD8B
p=2.59e-08
Log2 of CD3E
p=9.69e-07
Log2 of NCR1
p=2.68e-04
Log2 of CRTAM
p=8.28e-05
Log2 of EOMES
p=9.38e-03
Log2 of TBX21
p=2.10e-04
Log2 of GZMA
p=2.22e-03
Log2 of PRF1
p=5.57e-13
Log2 of TNF
p=0.400
Log2 of TOX
p=0.061
Log2 of FOXP3
p=1.90e-08
Log2 of CD68
p=1.17e-03
Log2 of CD163
p=1.61e-13
Log2 of PD-L1
p=0.736
Log2 of PD1
p=4.53e-08
Log2 of CD1C
p=6.9e-06
Log2 of LAMP3
p=0.739
Log2 of CD19
p=0.792
Log2 of CD79A
LR
HR

Fig. S3. Low-risk neuroblastomas exhibit the overall higher immune activity than high-risk neuroblastomas. Expression levels of representative immune response genes in low-risk neuroblastomas and high-risk neuroblastomas were examined in Cohort 1. The immune cell contents in the low-risk group, indicated by the expression level of *CD45* (a marker for hematopoietic cells), are greater than those in the high-risk counterpart. The overall immune activation status, indicated by the expression of *CD69* and *CD38* (immune activation markers) and *NKG7* (a CTL activation marker) in low-risk neuroblastoma was also higher than that in high-risk neuroblastomas. Among three major CTL subsets (CD8+ T, CD4+ T and NK cells, represented by *CD8B*, *CD4, CD3E* and *NCR1*, respectively), *CD4* expression was the highest, followed by *CD8B* and *NCR1* expression. The expression of CTL signature genes (*EOMES*, *TBX21, CRTAM, PERF1* and *GZMA*) was also higher in low-risk neuroblastomas than that of high-risk neuroblastomas. In contrast, markers of T cell exhaustion (*TOX*) and Treg (*FOXP3)* showed no difference in their expression between the two subsets. Expressions of relevant genes for macrophages (*CD68*, *CD163*, *PD-L1*, *PD-1*), dendritic cells (*CD1C*, *LAMP3*) and B cells (*CD19*, *CD79A*) were also examined in both low-risk and high-risk subsets. The expression unit of genes in Cohort 1 is Reads Per Million (RPM). LR: low-risk; HR: high-risk. The same analysis could not be done on Cohort 2 due to the setting in the R2 platform, but an equivalent result to Cohort 1 was obtained in the third cohort (Westerman-579) (http://r2.amc.nl).

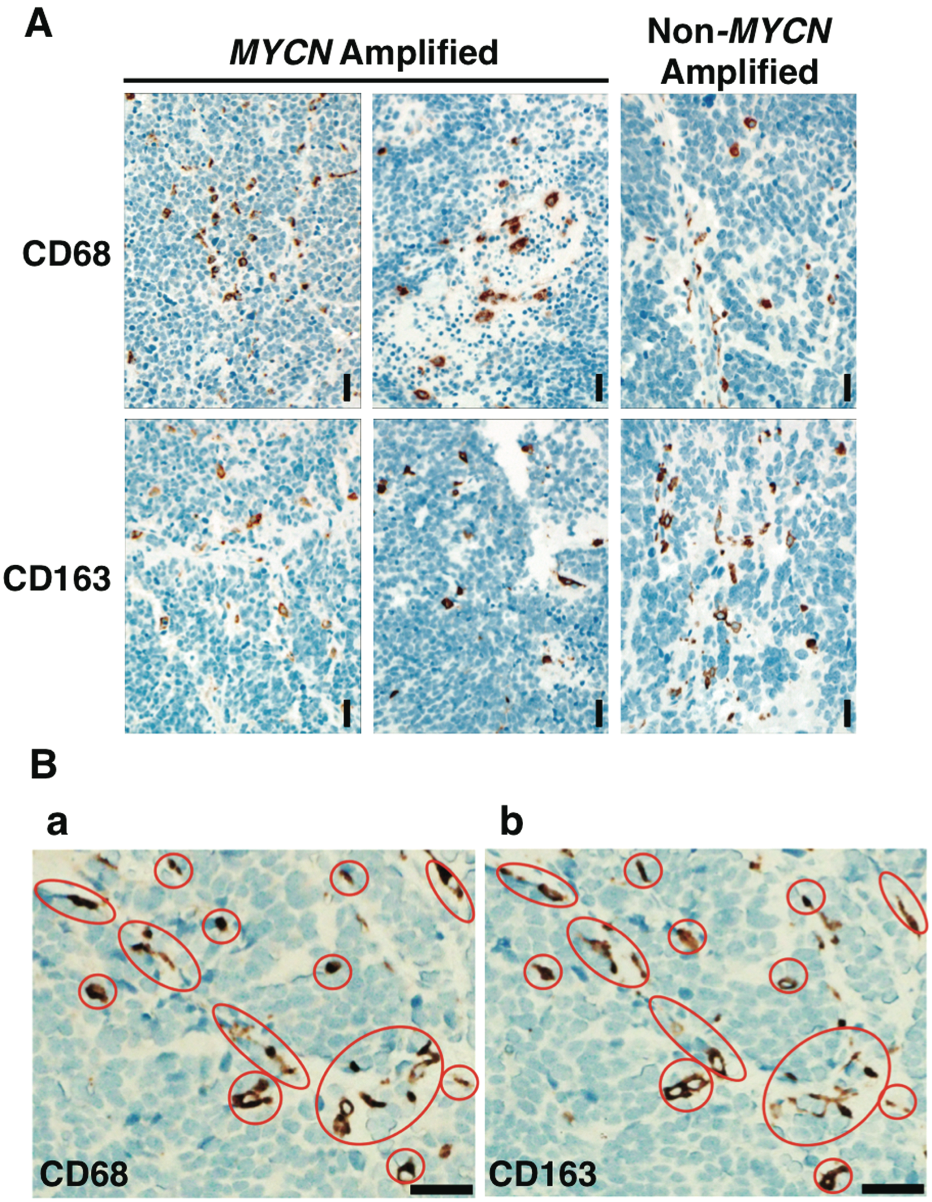

Fig. S4. (**A**) Immunohistochemical detection of CD68+ and CD163+ macrophages in neuroblastoma tissues. Representative high-risk neuroblastoma specimens (both *MYCN*-amplified and non-amplified cases) were subjected to immunohistochemical analysis using the corresponding antibodies. Readily detectable levels of CD68+ and CD163+ macrophages were seen in the tumor tissues, and the density and distribution of CD68+ and CD163+ macrophages in the tumor tissues were similar. The scale bar represents 20μm. (**B**) The majority of tumor associated macrophages in favorable histology neuroblastoma co-expresses CD68 and CD163. Serial sections from a favorable histology neuroblastoma were stained with either (**a**) anti-CD68 or (**b**) anti-CD163 antibodies. Red circles indicate macrophages that co-express CD68 and CD163. The scale bar: 50μm.
