# Supplementary material for: Macrophage-mediated anti-tumor immunity against high-risk neuroblastoma
Source: Genes Immun. 2022 May 7;23(3-4):129–40. doi: 10.1038/s41435-022-00172-w (PMC9232393; doi:10.1038/s41435-022-00172-w)
Supplement: Supplementary file 3 — Supplementary Table S1 [file 41435_2022_172_MOESM3_ESM.pdf]

| **Table S1. Biological pathways associated with *SLAMF7* expression in high-risk neuroblastomas of Cohort 1** | |
|---|---|
| **Pathway** | **p** |
| Cytokine_cytokine_receptor_interaction | 2.4e-44 |
| Osteoclast_differentiation | 1.4e-22 |
| Staphylococcus_aureus_infection | 6.4e-21 |
| Hematopoietic_cell_lineage | 1.4e-20 |
| Intestinal_immune_network_for_IgA_production | 1.6e-20 |
| Rheumatoid_arthritis | 1.2e-19 |
| Chemokine_signaling_pathway | 1.3e-19 |
| NF_kappa_B_signaling_pathway | 8.4e-18 |
| Leishmaniasis | 2.1e-17 |
| Graft_versus_host_disease | 2.5e-17 |
| Antigen_processing_and_presentation | 2.0e-16 |
| Lysosome | 4.2e-16 |
| Phagosome | 1.4e-15 |
| Allograft_rejection | 3.2e-15 |
| Cell_adhesion_molecules__CAMs_ | 3.6e-15 |
| Inflammatory_bowel_disease__IBD_ | 4.5e-15 |
| Tuberculosis | 5.9e-13 |
| TNF_signaling_pathway | 1.4e-12 |
| Natural_killer_cell_mediated_cytotoxicity | 2.4e-12 |
| Type_I_diabetes_mellitus | 1.7e-11 |
| Focal_adhesion | 1.9e-11 |
| Jak_STAT_signaling_pathway | 3.0e-11 |
| Toxoplasmosis | 5.0e-11 |
| Malaria | 1.7e-10 |
| ECM_receptor_interaction | 3.7e-10 |
| Primary_immunodeficiency | 3.8e-10 |
| Amoebiasis | 6.9e-10 |
| Leukocyte_transendothelial_migration | 7.2e-10 |
| B_cell_receptor_signaling_pathway | 8.3e-10 |
| Influenza_A | 3.9e-09 |
| Toll_like_receptor_signaling_pathway | 5.5e-09 |
| Viral_myocarditis | 1.2e-08 |
| Pertussis | 1.6e-08 |
| AGE_RAGE_signaling_pathway_in_diabetic_complications | 1.8e-08 |
| Asthma | 2.9e-08 |
| T_cell_receptor_signaling_pathway | 4.1e-08 |
| Platelet_activation | 5.8e-08 |

| | |
|---|---|
| Herpes_simplex_infection | 1.5e-07 |
| Chagas_disease__American_trypanosomiasis_ | 2.8e-07 |
| Measles | 3.6e-07 |
| HTLV_I_infection | 3.7e-07 |
| NOD_like_receptor_signaling_pathway | 3.8e-07 |
| Salmonella_infection | 6.2e-07 |
| Fc_gamma_R_mediated_phagocytosis | 1.7e-06 |
| Autoimmune_thyroid_disease | 2.5e-06 |
| Complement_and_coagulation_cascades | 3.5e-06 |
| Transcriptional_misregulation_in_cancer | 6.2e-06 |
| PI3K_Akt_signaling_pathway | 7.8e-06 |
| African_trypanosomiasis | 1.1e-05 |
| Acute_myeloid_leukemia | 1.3e-05 |
| Regulation_of_actin_cytoskeleton | 1.6e-05 |
| Legionellosis | 3.4e-05 |
| Pathways_in_cancer | 3.7e-05 |
| Proteoglycans_in_cancer | 5.4e-05 |
| Protein_digestion_and_absorption | 1.4e-04 |
| Apoptosis | 1.5e-04 |
| Other_glycan_degradation | 1.1e-03 |
| MAPK_signaling_pathway | 1.8e-03 |
| Cytosolic_DNA_sensing_pathway | 1.9e-03 |
| ‘Epstein_Barr_virus_infection | 1.9e-03 |
| VEGF_signaling_pathway | 2.4e-03 |
| Endocytosis | 3.3e-03 |
| Inflammatory_mediator_regulation_of_TRP_channels | 3.7e-03 |
| Ras_signaling_pathway | 3.9e-03 |
| Shigellosis | 4.6e-03 |
| Arrhythmogenic_right_ventricular_cardiomyopathy__ARVC_ | 4.8e-03 |
| Epithelial_cell_signaling_in_Helicobacter_pylori_infection | 6.9e-03 |
| Fc_epsilon_RI_signaling_pathway | 6.9e-03 |
| Hepatitis_B | 8.3e-03 |
| RIG_I_like_receptor_signaling_pathway | 9.2e-03 |
