# Supplementary material for: Macrophage-mediated anti-tumor immunity against high-risk neuroblastoma
Source: Genes Immun. 2022 May 7;23(3-4):129–40. doi: 10.1038/s41435-022-00172-w (PMC9232393; doi:10.1038/s41435-022-00172-w)
Supplement: Supplementary file 4 — Supplementary Table S2 [file 41435_2022_172_MOESM4_ESM.pdf]

**Table S2. Biological pathways associated with *SLAMF7* expression in high-risk neuroblastomas of Cohort 2**	
**Pathway**	**p value**
Cytokine_cytokine_receptor_interaction	4.0e-33
Staphylococcus_aureus_infection	5.0e-27
Graft_versus_host_disease	1.9e-25
Hematopoietic_cell_lineage	1.2e-24
Rheumatoid_arthritis	6.0e-24
Phagosome	2.0e-23
Osteoclast_differentiation	5.0e-23
Leishmaniasis	7.5e-23
Cell_adhesion_molecules__CAMs_	2.0e-22
Allograft_rejection	6.5e-22
Antigen_processing_and_presentation	9.5e-22
Intestinal_immune_network_for_IgA_production	3.0e-21
NF_kappa_B_signaling_pathway	4.0e-21
Inflammatory_bowel_disease__IBD_	7.1e-20
Tuberculosis	2.5e-18
Natural_killer_cell_mediated_cytotoxicity	1.9e-16
Type_I_diabetes_mellitus	6.0e-16
Malaria	8.4e-15
Chemokine_signaling_pathway	7.4e-14
Influenza_A	1.4e-13
Lysosome	1.5e-13
Viral_myocarditis	2.5e-13
Autoimmune_thyroid_disease	2.8e-13
Toxoplasmosis	2.9e-13
Primary_immunodeficiency	5.4e-12
Asthma	1.9e-11
Salmonella_infection	3.6e-11
Measles	4.9e-11
Leukocyte_transendothelial_migration	1.3e-10
Herpes_simplex_infection	2.8e-10
ECM_receptor_interaction	7.7e-10
TNF_signaling_pathway	1.2e-09
Toll_like_receptor_signaling_pathway	1.8e-09
T_cell_receptor_signaling_pathway	4.2e-09
Pertussis	4.8e-09
HTLV_I_infection	8.8e-09
Focal_adhesion	1.4e-08

NOD_like_receptor_signaling_pathway	1.4e-08
Amoebiasis	5.8e-08
Fc_gamma_R_mediated_phagocytosis	8.4e-08
B_cell_receptor_signaling_pathway	2.7e-07
Jak_STAT_signaling_pathway	4.0e-07
Chagas_disease__American_trypanosomiasis_	7.7e-07
Complement_and_coagulation_cascades	8.0e-07
Legionellosis	1.1e-05
AGE_RAGE_signaling_pathway_in_diabetic_complications	1.5e-05
Transcriptional_misregulation_in_cancer	1.7e-05
Platelet_activation	2.0e-05
Systemic_lupus_erythematosus	2.2e-05
African_trypanosomiasis	2.3e-05
Epstein_Barr_virus_infection	2.4e-05
Pathogenic_Escherichia_coli_infection	4.6e-05
PI3K_Akt_signaling_pathway	5.1e-05
Cytosolic_DNA_sensing_pathway	1.1e-04
Regulation_of_actin_cytoskeleton	1.3e-04
MAPK_signaling_pathway	2.7e-03
Hepatitis_C	2.9e-03
Shigellosis	2.9e-03
Fc_epsilon_RI_signaling_pathway	5.0e-03
Pathways_in_cancer	5.3e-03
Proteoglycans_in_cancer	7.5e-03
Hepatitis_B	7.7e-03
Epithelial_cell_signaling_in_Helicobacter_pylori_infection	8.2e-03
